# Severe Burn of the Eye from the Contents of a Fluid Core Golf Ball

**Published:** 1913-03

**Authors:** 


Severe Burn of the Eye from the Contents of a Fluid Core Golf Ball. W. O. Nance of Chicago (*Jour. Oph. and Oto-Laryn.*, Nov., 1912), reports the following case: Three weeks before a young man was removing the cover of a golf ball. The patient was leaning over his shoulder when suddenly a stream of so-called "acid" squirted up from the core of the ball and struck her directly in the eye. She suffered intense pain and the skin surrounding the eye was burned for a distance of one to two inches. There was a deep opacity of the cornea in almost its entire area. Vision was reduced to mere perception of light. He advised enucleation. The details of this case and the histories of others reported suggest to us as physicians the obligation we owe the public to warn them of the dangers of the fluid core ball when opened.

